# Universal Implementation of Newborn Screening in India

**DOI:** 10.3390/ijns6020024

**Published:** 2020-03-25

**Authors:** Thomas Mookken

**Affiliations:** NeoGen Labs, UCF Center, 84/3 Oil Mill Road, Lingararajpuram, Bengaluru 560 084, Karnataka, India; mookken@neogenlabs.com

**Keywords:** inborn errors of metabolism, neonatal, newborn screening, population screening, sickle cell disease

## Abstract

Newborn screening is a successful program in many developed countries. In India, the benefits of dried blood spot screening have been recognized and that screening is slowly gaining traction. There are significant issues standing in the way of universal implementation of a newborn screening program in India: awareness, cost, advocacy, public policy, and politics. Three regional screening programs, Chandigarh, Goa, and Kerala could serve as models for other programs in India. The data for this commentary were based on personal experiences from managing public newborn screening programs, searches on PubMed and Google, and personal interactions with experts in the field. The overwhelming recommendation is to universally screen for congenital hypothyroidism in India, because it is easy and inexpensive to treat, with excellent outcomes. It would also be beneficial to consider screening universally for glucose-6-phosphate dehydrogenase deficiency due to its high incidence and ease of treatment. Finally, sickle cell disease should be screened in those areas in India where it is prevalent due to the costs associated with universal screening. Achieving universal screening is a challenge, and it is very difficult to predict when every baby born in India will be screened for at least congenital hypothyroidism.

## 1. Introduction

Population screening in India is a challenge for a variety of reasons. “The Complex Promise of Newborn Screening” by Dr. Fiona Miller, presented in 2007, documents the reasons why [1]. Today, nearly all the hurdles presented in that paper are still relevant, including the following:

- The newborn screening (NBS) programs can make only a minor contribution to reducing the global burden of infant morbidity and mortality;
- Access to healthcare for the poor, particularly in rural areas, is challenging, and even the urban middle classes may experience serious problems in making good use of NBS test results;
- Neonatal care units, especially at the district level, are limited in their availability, with most neonatal care available through specialized tertiary units in urban areas; and,
- Prevalent “misunderstandings” of screening results may prove resistant to typical educational interventions, as interpretations of disease and disease risks are open to cultural influence.

Presently in India, there are no debates in the medical community questioning the benefits of screening; the consensus is that all babies need to be screened, but there is no coherent national strategy for implementing a universal screening program nor guidance on which disorders should be included in the screening panel.

In 2011, the National Neonatology Forum (NNF) recommended congenital hypothyroidism (CH), congenital adrenal hyperplasia (CAH), and glucose-6-phosphate dehydrogenase (G6PD) deficiency as the screening panel to implement for newborn screening in India [2].

Today, most babies born in India are not screened at all. Based on personal experience, I estimate that less than 750,000 babies born in India in 2019 were screened for at least one disorder (<3% of births).

### 1.1. Evolution of NBS Screening Tests in India

In 2004, Lal Path Labs, New Delhi, started offering screening tests for a comprehensive panel of disorders with the commissioning of a tandem mass spectrometer (MS/MS) [3]. This was the first organization in India to offer screening for a wide range of inborn errors of metabolism (IEMs). They were pioneers in offering commercial NBS services, but the market, unfortunately, was not ready for it. 

In 2007, the National Institute of Mental Health and Neurosciences (NIMHANS), an institution run by the Government of India, started offering screening for disorders by tandem mass spectrometry [4]. The hospitals and physicians who were aware of inborn errors of metabolism (IEMs) started using these services for diagnosis as opposed to screening and, therefore, did not achieve the benefits associated with pre-symptomatic treatment.

Today, there are numerous NBS laboratories, public and private, in India offering NBS tests. Some of them offer comprehensive NBS panels, resembling the Recommended Universal Screening Panel (RUSP) in the US or a subset of it [5]. Many, but not all, of them participate in the Newborn Screening Quality Assurance Program (NSQAP) offered by the Centers for Disease Control and Prevention (CDC), US [6].

### 1.2. Issues Impeding Universal Implementation of Newborn Screening Programs

Reviewing the principles of Wilson and Jungner’s screening criteria and applying it to NBS in India, the biggest impediments to starting programs are as follows [7]:

- The cost of case finding (including diagnosis) should be economically balanced to possible expenditure on medical care as a whole; and
- Facilities for diagnosis and treatment should be available.

NBS must be considered in the context of competing national healthcare priorities in India. In a country where many people live in extreme poverty (176 million at USD 1.90 or less per capita per day) and access to basic healthcare can be a challenge, where would NBS rank in priority? The answer is, “Not very high” [8].

The 2019 Indian health budget of approximately USD 9 billion allocates USD 6.50 per capita per year [9]. The total cost to perform one screening test for CH, as an example, on one baby on a dried blood spot (DBS) using an enzyme-linked immunosorbent assay (ELISA) is about USD 5.00, summarized in Table 1. This cost also includes resampling for testing unsuitable samples and overheads (rent, electricity, administration, maintenance, and depreciation) based on the experience of running an NBS laboratory [10]. Additional tests are approximately USD 1.50 each using ELISA.

Assuming all 27 million babies born in a year are screened for CH, the cost is 135 million USD, which is equivalent to 1.2% of the health budget, a significant cost in India. The costs decrease when additional tests are added due to shared consumables and logistics, but the high cost of screening the first disorder can be a barrier to starting a program. The customs duties and taxes levied on equipment and consumables are also substantial (as high as 32%), increasing screening costs.

There is no reliable incidence data to make the case for universal screening. There were only a few regional pilot programs over the years that attempted to measure the incidence of various inborn errors of metabolism (IEMs). The data collected from these have resulted in no large population screening programs.

There is a lack of awareness of IEMs in the medical community. Feedback received from physicians, when NeoGen Labs was introducing NBS in 2007, indicated the lack of emphasis or complete absence of learning of IEMs and their treatments when the physicians were in medical school. Due to this, IEM cases were often misdiagnosed, and unexplained deaths were attributed to sepsis, infection, sudden infant death syndrome, or other causes. An increasing number of Indian physicians are now being trained in western countries where NBS is routine, therefore, expertise has grown and physicians are now able to diagnose and treat these disorders. 

India has also lacked a recognized champion who was able to advocate for NBS and had the power to make it a universal benefit in India, comparable to what Dr. C. Padilla has been able to accomplish in the Philippines [11]. There have been NBS initiatives announced over the years in the public sphere, but many are yet to be realized.

The above is not an exhaustive list of problems facing NBS in India but if the Government of India’s Ministry of Health and Family Welfare addresses these issues, it will help the cause of universal screening in India.

### 1.3. Current State of Universal Screening

At the highest levels of the national government, the benefits of NBS have not been fully understood and, as a result, no national policy on NBS has been created. Parent advocacy groups, like those in the United States, who have the resources to make a case for funding of screening at the national or state levels, even though the benefits of NBS are well understood by the medical community, are largely absent. Metabolic Errors and Rare Diseases (MERD) India Foundation is the only private organization making a case for universal screening in India and has done a commendable job with limited resources.

#### 1.3.1. Private NBS Programs

Private hospitals have taken the lead in NBS in India, most of which have physicians on their staff who have been exposed to NBS while training abroad. From a competitive view point, NBS differentiates their service offerings and generates an additional income stream. Most of these are in urban areas with an affluent clientele who understand the benefits of screening. The panels range from three disorders to a comprehensive set (50+) that mirrors the RUSP, widely followed in the US [10]. 

#### 1.3.2. Public NBS Programs

Screening programs in public hospitals have the potential to achieve universal screening. About 52% of the births in India are in public hospitals where the cost of delivery is less than $60 [12]. Since the cost of services in these hospitals, including NBS tests, are free, it is possible to screen all babies born in public hospitals.

In this commentary, I discuss three public screening programs with varying degrees of complexity (panels, geographical areas covered, and births screened per year) that have been running for more than 5 years. Each of these programs follow different models in their implementation. 

The states where these programs are implemented also have low infant mortality rates (IMR), well below the Indian average of 33 in 2017 [13]. In 2017, the IMR for Chandigarh was 14, Goa was 9, and Kerala was 10. In India, the three leading causes of infant mortality are (a) prematurity and low birth weight, (b) neonatal infections, and (c) birth asphyxia and birth trauma [14]. Even though the IMR rate has come down significantly from 53 in 2008, these are still the leading causes of death [15].

Chandigarh’s NBS program is concentrated in four urban government hospitals, screening about 15,000 births per year [16]; Goa screens approximately 12,500 births per year [10] in 13 government hospitals, and Kerala screens more than 140,000 births per year in over 90 government hospitals [17]. None of these programs screen births in private hospitals. 

All these programs screen for panels of disorders that are well understood by physicians in India and easily treatable. Disorders screened by MS/MS (fatty acid oxidation disorders, organic acid disorders, and amino acid disorders) are not part of the screening panels due to resource constraints (significant capital costs, few experts, lack of treatment facilities, and high cost of diets).

There are also other NBS programs, but these three are unique in their longevity. In each of these programs, the institutional birth rates for the regions covered by the programs are over 95%, which makes the task of universal screening easier. All these programs offer free screening for births in the government hospitals.

It is important to note that none of these public programs were started based on the results of pilot programs that were then translated into public health policy based on the benefits of screening. They were started for other reasons, identified in the program descriptions detailed below. 

##### The Chandigarh Program

In 2007, the union territory (UT) of Chandigarh in India started a program to study the prevalence of three disorders (CH, CAH, and G6PD deficiency) in the territory [18]. 

This effort has evolved into the pioneering public NBS program in India and continues to this day with the addition of other government hospitals in the UT and the testing of additional disorders. The success of the program is based on a team that is passionate about NBS, a small number of births (~15,000 in the public hospitals in 2016), close to 100% institutional births, and a small geographical area that optimizes logistic efficiency [16].

The screening tests are performed in the NBS laboratory operated by the Government of Chandigarh. The laboratory participates in NSQAP.

##### The Goa Program

The Goa 1.0 NBS Program (2008 to 2013) was initiated based on the desire of the state government to improve neonatal care [10]. Since health policy in India has an emphasis on IMR and incentivizes reducing it, it was believed that NBS could be a factor in improving this statistic in Goa [18]. The NBS program screened every baby born in a public hospital (~48,000), about 50% of the births in Goa in the five-year period.

The disorders selected were a comprehensive panel of more than 50 disorders. This program followed a public–private partnership (PPP) model that was financially beneficial to the state government since their investment was minimal. All aspects of the program, other than sample collection, through the delivery of the screening report were handled by the PPP. The program laboratory participated in NSQAP. Follow-up and treatment were the responsibility of the state government [19].

The program was successful in identifying disorders in Goa and raising NBS awareness in India. It also identified issues that needed to be overcome for a successful NBS program run by any state government in India. Subsequent public programs have referred to the Goa NBS program to justify screening initiatives in their states [20].

The program also pointed out shortcomings in an NBS program, primarily in follow up and treatment resources (both in expertise and in availability and access to diets). One of the disappointments was there were not many success stories to showcase the benefits due to the lack of a treatment infrastructure. In 2013, the program was terminated for political reasons with a change in the government. Nevertheless, this program is a precursor to a successful universal screening program in India.

The Goa 2.0 NBS Program started in Aug 2018, incorporating the learnings from Goa 1.0. All births in government hospitals are screened and, once again, follow the PPP model [21]. The panel was reduced to six disorders (CH, CAH, G6PD, galactosemia (GALT), biotinidase, and cystic fibrosis). There are adequate resources to treat these disorders. High-risk deliveries and all neonatal intensive care unit (NICU) admissions are screened for over 50 disorders, including those by MS/MS. 

The shortcomings of the previous program were addressed, and more emphasis was placed on follow-up activities, access to experts, and availability of diets. Political will ensures the success of the program, and its progress is monitored at the highest levels of government. 

##### The Kerala Program

The Public Health Laboratory in Kerala submitted a proposal to the central government for a pilot NBS program in 2011, which was funded [2]. The program was launched in 2012, screening for four disorders. Since then, the program has grown and aimed to screen all births (~140,000) in government hospitals in 2018 [17]. The program screens for CH, CAH, G6PD, and GALT in four laboratories spread across the state. None of the state screening laboratories participate in NSQAP.

After the program meets the goal of screening all the births in the government hospitals (25% of all births in Kerala per year), the program plans to extend screening to the private hospitals, which account for the remaining 75% of the births (~400,000). In the upcoming phase of the NBS program, 300,000 births per year in the next two years, are planned to be screened [22].

The program is streamlining the collection and transport of samples. The testing infrastructure is in place and samples are processed in a reasonable timeframe. Even though the expertise is available to treat affected babies, the communication of positive results, follow-up, and treatment are areas that need to improve [23]. It will take time to work out the shortcomings in the program, but Kerala is the best positioned among all the large states in India to implement a universal screening program.

### 1.4. Disorders to be Screened for in a Universal Screening Program 

The conclusions from public studies recommend a universal screening program should include two to four disorders [2,18,24]. Given the large number of births in India per year, and the costs involved, if only one disorder is to be screened, it should be CH. Dr. Bradford Therrell succinctly summarized the reasons on the choice of CH in an editorial in Indian Pediatrics: CH is a disorder that is well understood, easy to detect, and inexpensive to treat. From a financial point of view, the return on investment is very high [25].

In an article in 2005, Dr. N B Kumta estimated about 1.5% of the babies born in India every year are affected by G6PD deficiency [26]. Preliminary data from the Goa 2.0 program indicated an incidence of 0.36% from a sample size of 10,000 [9]. Some tribal populations in India with sickle cell anemia also have a prevalence of G6PD deficiency [27]. Testing for G6PD deficiency should also be considered for universal screening in India.

India has a large tribal population, amounting to 8.6% of the total population in 2011 [27]. These tribal populations have a disproportionately higher prevalence of the sickle gene (up to 40% in certain geographies). Based on this data, targeted screening of these at-risk populations for hemoglobinopathy variants (including sickle cell disease) should also be included in a screening program. Sickle cell disease is well understood, has a treatment, and has significant funding from the Government of India.

## 2. Steps to Universal Screening

### 2.1. Awareness and Policy

With a few limited population screening programs currently operating in India, the awareness of the benefits of NBS is increasing. Currently, there is no clear national policy on NBS, but the increasing awareness could spur the creation of one. India has a federal system of government, like the United States, and health is a state subject. Since the funding for health is largely from the central government, they have the power to drive the implementation of NBS in the public sector with a recommended panel of disorders.

It is unlikely that the government can afford to bear all the costs associated with a universal screening program, and the program will have to be done in partnership with the private hospitals. In private hospitals, if parents request screening, they will have to pay for it. This is an impediment in achieving the universal screening goal since parents may decline screening due to costs and a lack of awareness of the benefits of NBS.

### 2.2. Service Model to Achieve Universal Newborn Screening

There are two models that could be followed: point of care (POC) and central laboratory (CL).

If only CH is being screened, it would be more efficient and economical to screen in the hospital laboratory. Logistic complexity will be minimal and results available quickly, possibly before the baby is discharged. Once discharged from the hospital, especially in a government hospital, it is difficult to track patients down for follow up if the screen is positive.

In India, many births are in private birthing facilities with very basic laboratory infrastructure, if any. In these cases, it would be advantageous to use a CL. Also, if more than one disorder is screened, a CL would be preferred. While logistics of shipping samples to the CL will impact the turnaround time for results, a CL with a focus on NBS will have more experience and efficiency in the overall screening process.

### 2.3. Operations Model

In the public sector, the smaller states could follow a PPP model where the private partner is responsible for all or some aspects of the program obviating the need for the state to invest in building the capability.

In larger states, there will be a requirement for multiple CLs due to the number of births (9 out of 29 states in India have more than one million births per year) and logistics due to the large geographical areas covered. In these cases, the NBS programs are best served by the state taking the lead [28]. If the state governments invest in developing infrastructure and capabilities, the programs are less likely to be subject to the vagaries of politics when governments change.

In India, public healthcare serves as an option of last resort, with a few exceptions. Those who can afford private healthcare will always choose it. Therefore, the private labs will play an important role in NBS for those willing to pay for service.

## 3. Realities in Implementing a Public Program

Only when the policies and funding are in place should a program be implemented. Depending on which model is used, challenges abound in infrastructure, bureaucracy, training, and sample collection, which need to be overcome. 

A critical message to all stake holders is that “NBS is a program and does not end with a result of a screening test”. They have to recognize that NBS is different from routine laboratory testing, and it requires a different infrastructure. Screening is just the first step, and it is imperative that a program is in place to ensure success. First and foremost is that the primary goal of the public program is to deliver a public health screening service to benefit the baby. Programs should be held accountable for their actions, especially timeliness, accuracy, and errors. 

Today, many of the processes for vaccinations are already in place at the public hospitals. These processes can serve as models for DBS collection in these hospitals so that no babies are missed. An opt-out policy is better than opt-in at the public hospitals, since explaining and getting consent can be time consuming and expensive. In the private hospitals, opt-in would be the preferred method, since it is a fee-for-service model.

The screening laboratories must participate in external quality assurance schemes (NSQAP) and adhere to standards and quality accreditations with the goal of getting a timely, accurate result. These must be incorporated at the start of the program for its success. One of the challenges currently is the availability of assessors in India with expertise in NBS for ISO 15189 accreditation, which needs to be addressed.

The key to success of the program is the continuous follow up with the affected babies. There are cases (especially for births in public hospitals) where the affected baby’s health improves and the therapy is discontinued by the parents without the knowledge of the treating physicians. This defeats the purpose of the screening program.

India has a network of Accredited Social Health Activist (ASHA) workers who provide help in accessing health services, and their services could be leveraged for follow-up activities [29]. They are trained professionals, primarily women, with a focus on women and child healthcare, and come from the rural communities they serve. The ASHA workers can help in monitoring cases to ensure compliance and bring patients to the notice of the physicians, if required.

## 4. Conclusions

A universal implementation of newborn screening is a challenge in India, but the increasing awareness and programs over the past decade have led to more babies being screened every year. The results of the existing programs suggest to the policy makers in India that there is a benefit in implementing a universal NBS programs. The challenges faced in India, especially financial, make it very difficult to predict when every baby born in India will be screened. In my opinion, it will be at least another decade. 

## Figures and Tables

**Table 1:** Costs for performing a congenital hypothyroidism (CH) screen.

Item	Cost (USD)
Sample Collection Consumables	1.00
Sample Transport and Logistics	0.50
CH Assay	1.00
Laboratory Consumables	0.50
Labor	1.00
Overhead	1.00
**Total Cost for CH**	**5.00**
Screening an Additional Disorder	1.50
